# Histone methylation levels correlate with TGFBIp and extracellular matrix gene expression in normal and granular corneal dystrophy type 2 corneal fibroblasts

**DOI:** 10.1186/s12920-015-0151-8

**Published:** 2015-11-09

**Authors:** Yong-Sun Maeng, Ga-Hyun Lee, Seung-Il Choi, Kyu Seo Kim, Eung Kweon Kim

**Affiliations:** Department of Ophthalmology, Corneal Dystrophy Research Institute, Yonsei University College of Medicine, 250 Seongsanno, Seodaemun-gu, Seoul 120-752 South Korea; Emory University School of Medicine, Atlanta, GA USA; Institute of Vision Research, Severance Biomedical Science Institute, Brain Korea 21 Plus Project for Medical Science, Yonsei University College of Medicine, Seoul, South Korea

**Keywords:** GCD2, TGFBIp, Extracellular matrix, Histone methylation

## Abstract

**Background:**

TGFβ1-induced expression of transforming growth factor β-induced protein (TGFBIp) and extracellular matrix (ECM) genes plays a major role in the development of granular corneal dystrophy type 2 (GCD2: also called Avellino corneal dystrophy). Although some key transcription factors are known, the epigenetic mechanisms modulating TGFBIp and ECM expression remain unclear. We examined the role of chromatin markers such as histone H3 lysine methylation (H3Kme) in TGFβ1-induced TGFBIp and ECM gene expression in normal and GCD2-derived human corneal fibroblasts.

**Methods:**

Wild-type (*n* = 3), GCD2-heterozygous (*n* = 1), and GCD2-homozygous (*n* = 3) primary human corneal fibroblasts were harvested from human donors and patients prepared. Microarray and gene-expression profiling, Chromatin immunoprecipitation microarray analysis, and Methylated DNA isolation assay-assisted CpG microarrays was performed in Wild-type and GCD2-homozygous human cells.

**Results:**

Transcription and extracellular-secretion levels of TGFBIp were high in normal cells compared with those in GCD2-derived cells and were related to H3K4me3 levels but not to DNA methylation over the TGFBI locus. TGFβ1 increased the expression of TGFBIp and the ECM-associated genes connective tissue growth factor, collagen-α2[Ι], and plasminogen activator inhibitor-1 in normal corneal fibroblasts. Increased levels of gene-activating markers (H3K4me1/3) and decreased levels of repressive markers (H3K27me3) at the promoters of those gene accompanied the changes in expression. TGFβ1 also increased recruitment of the H3K4 methyltransferase MLL1 and of SET7/9 and also the binding of Smad3 to the promoters. Knockdown of both MLL1 and SET7/9 significantly blocked the TGFβ1-induced gene expression and inhibited TGFβ1-induced changes in promoter H3K4me1/3 levels. Those effects were very weak, however, in GCD2-derived corneal fibroblasts.

**Conclusions:**

Taken together, the results show the functional role of H3K4me in TGFβ1-mediated TGFBIp and ECM gene expression in corneal fibroblasts. Pharmacologic and other therapies that regulate these modifications could have potential cornea-protective effects for granular corneal dystrophy.

**Electronic supplementary material:**

The online version of this article (doi:10.1186/s12920-015-0151-8) contains supplementary material, which is available to authorized users.

## Background

Granular corneal dystrophy type 2 (GCD2, also called Avellino corneal dystrophy) is an autosomal dominant disorder caused by an arginine-to-histidine substitution at codon 124 (R124H) of the transforming growth factor β-induced gene (*TGFBI*) on chromosome 5q31.8. *TGFBI* encodes a protein of 683 amino acids (TGFBIp) [1, 2]. TGFBIp is an extracellular matrix (ECM) protein that is structurally homologous to the axon-guidance protein fasciclin I [3–5]. TGFBIp contains four tandem repeats of fasciclin I domains and an EMI protein-protein interaction domain and plays a key role in a variety of cellular responses including adhesion, migration, proliferation, angiogenesis, and wound healing [6–8]. The age-dependent, progressive accumulation of hyaline and amyloid-containing TGFBIp in the corneal stroma is a hallmark of GCD2, which is characterized by the production of TGFBIp in the corneal epithelia and stroma, interfering with corneal transparency [9]. Moreover, homozygous R124H mutations in *TGFBI* cause the severe phenotype of GCD2 characterized by early onset and confluent superficial opacity [10, 11]. The molecular mechanisms and cellular role of TGFBIp in corneal dystrophy pathogenesis are poorly understood.

TGFβ1 induces the progressive accumulation of TGFBIp and ECM proteins such as collagens, fibronectin, and keratin in the corneal epithelia and stroma [12–14]. TGFβ1 increases ECM accumulation through the induction of its downstream effector, connective tissue growth factor (CTGF) [15, 16], and by decreasing matrix degradation through the inhibition of proteases or the activation of protease inhibitors such as plasminogen activator inhibitor-1 (PAI-1) in glomerular mesangial cells [17]. TGFβ1 can regulate TGFBIp expression through Smad transcription factors and E-box-dependent mechanisms [18–22]. However, the subtle nuclear-chromatin mechanisms involved in the TGF-β1-induced expression of key ECM genes in corneal fibroblasts are not clear.

Gene regulation by extracellular stimuli involves not only transcription factors binding to their cognate DNA binding sites but also epigenetic changes in chromatin without alterations in DNA sequence. Post-translational modifications, including acetylation, methylation, and ubiquitination at key lysines, on the amino-terminal tails of nucleosomal histones such as H3 and H4 play a key role in modulating chromatin structure and gene transcription [23, 24]. They form a “histone code” that can dictate the transcriptional outcomes of gene activation or repression [25]. In general, the acetylation of H3 lysines (H3KAc) is associated with active gene transcription, whereas the methylation of H3 lysines (H3Kme) can be associated with either active or inactive gene promoters, depending on the position of the modified lysine. H3KAc is mediated by histone acetyl transferases, whereas H3Kme is mediated by histone methyltransferases (HMTs). HMTs can mono-methylate, di-methylate, or tri-methylate (H3Kme1, H3Kme2, or H3Kme3, respectively) specific lysine residues, thereby adding another epigenetic regulatory layer [24].

The methylation of the fourth lysine in H3 (H3K4me) is usually associated with gene activation and transcriptional elongation and is mediated by HMTs such as SET1, MLL1-4, and SET7/9 [24, 26–28]. H3K9me, on the other hand, is generally associated with gene repression and is mediated by HMTs such as SUV39H1, G9a, and SETDB1/ESET [26]. Other H3 lysines, including H3K27, H3K36, and H3K79, can also be methylated to various degrees [26]. In addition, the discovery of histone lysine demethylases has added another dimension to gene regulation [29]. Together, these factors create a fine balance of gene regulation, a disruption of which could result in abnormal gene expression and disease phenotypes.

It is not known whether lysine methylation in the H3 promoter plays a role in the TGFβ1-induced transcription of TGFBIp and ECM-associated genes in corneal fibroblasts. Here, we show that the transcription and ECM secretion levels of TGFBIp are high in normal cells compared with those in GCD2 cells and are related to H3K4me3 levels. We also show that TGFβ1 leads to the enrichment of H3K4me1/3 and the depletion of H3K27me3 markers on the promoters of TGFBIp and ECM-associated genes in corneal fibroblasts. Furthermore, H3K4, HMT, MLL1, and SET7/9 all seemed to play a role in the TGFβ1-induced expression of TGFBIp and ECM genes. These data show novel epigenetic mechanisms of TGFβ1 actions in corneal fibroblasts related to TGFBIp and ECM deposition and GCD2 pathogenesis.

## Methods

### Materials

Recombinant TGFβ1 was obtained from R&D Systems (Minneapolis, MN); normal rabbit IgG, normal mouse IgG, and ChIP-Grade Protein A/G Magnetic Beads were obtained from Thermo Scientific, (Rockford, IL, USA). The following antibodies were used in western-blot analyses and ChIP assays: anti-smad3 (ab28379), anti-SET7/9 (ab13731), anti-histone H3 (ab100938), anti-histone H3 trimethyl K9 (ab8898), and anti-H3 trimethyl K27 (ab1782) from Abcam (Cambridge, MA); anti-MLL1 (05–765), anti-H3 monomethyl K4 (07–436), and anti-H3 trimethyl K4 (07–473) from Millipore (Millipore Corp., Bedford, MA); anti-TGFBIp (AF2935) from R&D Systems; and anti-β-actin (A-5441) from Sigma-Aldrich (St. Louis, MO). MLL1 shRNA lentivirus (sc-38039-v), SET7/9 shRNA lentivirus (sc-44094-v), and control shRNA lentivirus (sc-108080) was purchased from Santa Cruz Biotechnology Inc. (Heidelberg, Germany).

### Isolation and culture of primary corneal fibroblasts

Wild-type (*n* = 2) and GCD2-homozygous (*n* = 2) (Arg124His mutation) primary human corneal fibroblasts were harvested from human donors (age matched) and patients prepared using a previously published method [30]. Donor confidentiality was maintained according to the Declaration of Helsinki, and the Severance Hospital IRB Committee (CR04124), Yonsei University approved the protocol. The wild-type and GCD2 cells were diagnosed by DNA sequencing of *TGFBI* mutations. After the removal of the corneal buttons from the donors for penetrating keratoplasty, the remaining corneal rims were harvested for culture of the corneal fibroblasts. The medical records of the donors from the eye bank of Yonsei University Severance Hospital did not show any genetic or systemic metabolic disease. The fibroblasts grown from the pieces of corneal rims were treated as healthy controls. The corneal fibroblast cells were cultured in DMEM supplemented with 10 % fetal bovine serum at 37 °C in a humidified incubator with 95 % air and 5 % CO2.

### Microarray and gene-expression profiling

Transcriptional profiles were evaluated in two independent cell preparations using a cDNA microarray (GeneChip Human Gene 1.0 ST Array, GeneChip; Affymetrix, Santa Clara, CA) containing approximately 29,000 genes. To determine the variation and average changes (*x*-fold) in the expression of different genes in each sample and to compare the samples, we analyzed four samples (normal sample pairs 1 and 2 (age matched) and GCD2-homozygous (Arg124His mutation) sample pairs 1 and 2) in an additional microarray analysis without total RNA pooling. Total RNA was extracted from the cells (TRIzol; Invitrogen, Carlsbad, CA), followed by purification (RNeasy kit; Qiagen, Valencia, CA) to remove residual DNA. The concentration of total RNA was determined by UV spectrophotometry (ND-1000 UV–vis Spectrophotometer; Nanodrop Technologies, Wilmington, DE). Two quality controls were used for each RNA sample: (1) an A_260_/A_280_ ratio between 1.7 and 2.3 and (2) an electropherogram showing two distinct ribosomal peaks corresponding to 18S and 28S RNAs, respectively, at a ratio of 28S/18S > 0.5 with minimal or no degradation.

100 ng of total RNA was used for the microarray experiment. GeneChip Human Gene 1.0 ST Arrays (Affymetrix, Santa Clara, USA) were used. Whole microarray experiment was performed according to the manufacturers protocol. The Affymetrix GeneChip Whole Transcript (WT) sense Target Labeling Assay is designed to generate amplified and biotynylated sense-strand DNA targets from the entire expressed genome. Protocol is optimized for the use with the GeneChip Sense Target (ST) Arrays, where the probes are distributed throughout the entire lenght of each transcript. At first 100 ng of total RNA was mixed with Poly-A RNA controls (GeneChip Eukaryotic Poly-A Control Kit, Affymetrix, Santa Clara, USA). Subsequently cDNA sysnthesis was performed with random hexamers tagged with a T7 promoter sequence (GeneChip WT cDNA Synthesis and Amplification Kit, Sub-kit 1: GeneChip WT cDNA Synthesis Kit; Affymetrix, Santa Clara, USA). The double-stranded cDNA was then used as a template to produce many copies of antisense cRNA (GeneChip WT cDNA Synthesis and Amplification Kit, Sub-kit 2: GeneChip WT cDNA Amplification Kit; Affymetrix, Santa Clara, USA). cRNA was treated with the cleanup procedure (GeneChip IVT cRNA, cDNA Cleanup Kit; Affymetrix, Santa Clara, USA). Subsequently cRNA yield was determined by spectrophotometric measurement (NanoDrop ND-1000; Thermoscientific). 10 μg of cRNA was then used in the second cycle of cDNA synthesis. The random hexamers were used to prime riverse transcription of the cRNA to produce single-stranded DNA in the sense orientation (GeneChip WT cDNA Synthesis and Amplification Kit, Sub-kit 1: GeneChip WT cDNA Synthesis Kit; Affymetrix, Santa Clara, USA). During this step of the procedure, in order to reproducibly fragment ssDNA, dUTP was incorporated. Subsequently, ssDNA was then proceed with the cleanup procedure (GeneChip IVT cRNA, cDNA Cleanup Kit; Affymetrix, Santa Clara, USA). DNA yield was determined by spectrophotometric measurement (NanoDrop ND-1000; Thermoscientific).

5 μg of single-stranded DNA was treated with a combination of two enzymes: uracil DNA glycosidase (UDG) and apurinic/apyrimidinic endonuclease 1 (APE 1), specifically recognizing the dUTP nucleotides and breaking the DNA strand (GeneChip WT Terminal Labeling Kit; Affymetrix, Santa Clara, USA). Subsequently, fragmented DNA was labeled by terminal deoxynucleotidyl transferase (TdT) with the DNA Labeling Reagent, that was covalently linked to biotin (GeneChip WT Terminal Labeling Kit; Affymetrix, Santa Clara, USA). Then fragmented and labeled DNA was hybridized with the GeneChip Human Gene 1.0 ST Array (Affymetrix, Santa Clara, USA). Apart from DNA, hybridization cocktail included: Eukaryotic Hybridization Controls (bioB, bioC, bioD, cre), Control Oligonucleotide B2 (GeneChip Hybridization Control Kit; Affymetrix, Santa Clara, USA), 2x Hybridization Mix, DMSO, water (GeneChip Hybridization, Wash and Stain Kit- Hybridization Module; Affymetrix, Santa Clara, USA). Hybridization was performed in 450C/ 60 rpm/ 17 h ±1 h at hybridization oven (GeneChip Hybridization Oven 640; Affymetrix, Santa Clara, USA).

After the hybridization, arrays were registered in GeneChip Operating Software (GCOS), and subsequently washed and stained with the use of GeneChip Fluidics Station 450 (Affymetrix, Santa Clara, USA) and FS450_0007 protocol (GeneChip Hybridization, Wash and Stain Kit-Stain Module; Wash Buffer A; Wash Buffer B; Affymetrix, Santa Clara, USA). After the wash protocol was finished arrays were scanned with the GeneChip Scanner 3000 7G (Affymetrix, Santa Clara, USA) controlled by GeneChip Operating Software. The raw signal intensities were normalized (GeneChip Operating Software [GCOS] algorithm; Affymetrix); and the data were analyzed (Gene Chip DNA Analysis Software [GDAS], ver. 2.0) according to the Affymetrix GeneChip Expression Analysis Technical Manual (http://www.affymetrix.com). We detected a twofold change in differential gene expression between the normal and GCD2-homozygous (Arg124His mutation) samples. Expression of ECM-associated genes (TGFBIp, COL1A2, CTGF, and PAI-1) and H3K4 methyltransferase genes (MLL1, MLL2, ASH1L, SMYD3, SET7/9 and SETMAR) is presented as the log2 ratio. For statistical analysis, we used a two-tailed, unpaired Student’s t-test (significance threshold *P* < 0.05) to assess differences between the two cell types.

### ShRNA infection

Lentiviruses containing human SET7/9, MLL1, and control shRNA, respectively, were purchased from Santa Cruz Biotechnology Inc. and used to infect corneal fibroblasts according to the manufacturer’s instructions. Infected cells were selected using puromycin.

### Real-time quantitative reverse transcription PCR

Total RNA was isolated from corneal fibroblasts by extraction in TRIZOL reagent (Invitrogen, Carlsbad, CA). Using the Power SYBR Green RNA-to-CT™ 1-Step kit (Applied Biosystems, Foster City, CA, USA) and StepOnePlus™ (Applied Biosystems, Foster City, CA, USA), mRNA expression of GAPDH, TGFBIp, collagen type I alpha 2 (COL1A2), plasminogen activator inhibitor-1 (PAI-1), and CTGF was measured according to the manufacturer’s instructions. The PCR conditions for all the genes were as follows: 48 °C for 30 min, 95 °C for 10 min, then 40 cycles of 95 °C for 15 s and 60 °C for 1 min. The results were based on cycle threshold (Ct) values. We calculated the differences between the Ct values for the experimental genes and GAPDH (a reference gene) and graphed the results as the ratio of each RNA level to the calibrator sample level. The primers used for gene amplification were the following: TGFBI, 5′-CACAGTCTTTGCTCCCACAA-3′ (sense) and 5′-CTCCGCTAACCAGGATTTCA-3′ (antisense); GAPDH, 5′-ATGGGGAAGGTGAAGGTCG-3′ (sense) and 5′-GGGGTCATTGATGGCAACAATA-3′ (antisense); COL1A2, 5′- ATGGCTACCCAACTTGCCTT-3′(sense) and 5′-ACAGCCTTTTTCAGGTTGCC-3′ (antisense); PAI-1, 5′-CTGTCATAGTCTCAGCCCGC-3′ (sense) and 5′-AAAGGACTGTTCCTGTGGGG-3′ (antisense); and CTGF, 5′-GTGCATCCGTACTCCCAAAA-3′ (sense) and 5′-ATCGGCCGTCGGTACATACT-3′(antisense). Three independent experiments were performed, and statistical analysis was carried out using Newman-Keuls multiple comparison tests.

### Chromatin immunoprecipitation for microarray analysis

Chromatin immunoprecipitation (ChIP) assays were performed using the EpiQuik TM Chromatin Immunoprecipitation Kit (Epigentek) based on the protocol provided by the supplier (Epigentek Group Inc., Brooklyn, NY). Briefly, samples containing 4 × 10^6^ human corneal fibroblasts were chemically cross-linked by the addition of fresh 11 % formaldehyde solution (1:10 volumetric ratio of solution to sample) for 15 min at room temperature. The cells were then rinsed twice with 1× PBS, resuspended in CP3 lysis buffer containing a protease-inhibitor cocktail, and sonicated until the cross-linked chromatin was sheared to an average DNA fragment length of 200–1000 bp. One per cent of the sonicated lysate was used to quantify the total amount of DNA present in the different samples before immunoprecipitation (input). The sonicated samples were immunoprecipitated with H3K4me3 (Millipore, Millipore Corp., Bedford, MA) and H3K27me3 (Abcam, Cambridge, MA) antibodies using the EpiQuik TM Chromatin Immunoprecipitation Kit (Epigentek). Cross-linking between DNA and proteins was reversed by heating the samples at 65 °C for 15 min followed by Proteinase K digestion at 65 °C for 1.5 h. After cleaning on spin columns, the DNA was eluted in 10 mM Tris–EDTA buffer.

### Chromatin immunoprecipitation microarray analysis

The immunoprecipitated and input DNA was amplified using a whole-genome amplification kit (GenomePlex® Complete Whole Genome Amplification Kit, Sigma, USA) according to the manufacturer’s instructions. The amplified samples were purified using the QIAQuick PCR clean-up kit (Qiagen). Labeling reactions were performed with 4 μg purified amplified DNA and a Bioprime labeling kit (Invitrogen) according to the manufacturer’s instructions in a volume of 50 μl with a modified dNTP pool containing 120 μM each of dATP, dGTP, and dCTP; 60 μM dTTP; and 60 μM Cy5-dUTP (for the immunoprecipitated sample) or Cy3-dUTP (for the input sample). Labeled targets were subsequently purified using the QIAQuick PCR clean-up kit (Qiagen). Then, the dye-labeled DNA samples were purified and quantified using an ND-1000 spectrophotometer (NanoDrop Technologies, Inc., Wilmington, DE). After checking the labeling efficiency, each sample containing 2.5–5.0 μg cyanine 3-labeled and cyanine 5-labeled DNA target was mixed and then resuspended with 2X hybridization buffer, Cot-1 DNA, Agilent 10X blocking agent, and de-ionized formamide. Before hybridization to the array, the 260-μl hybridization mixtures were denatured at 95 °C for 3 min and incubated at 37 °C for 30 min. The hybridization mixtures were centrifuged at 17,900 g for 1 min and directly pipetted onto the Human Promoter 1 M microarray (Agilent Technology, USA). The arrays were hybridized at 65 °C for 40 h using an Agilent Hybridization oven (Agilent Technology, USA). The hybridized microarrays were washed according to the manufacturer’s protocol (Agilent Technology, USA).

### Data acquisition and analysis

The hybridization images were analyzed using an Agilent DNA Microarray Scanner (Agilent Technology, USA), and the data were quantified using the Agilent Feature Extraction software (Agilent Technology, USA). The preprocessing of the raw data and the normalization steps were performed using the Agilent Genomic Workbench software according to the manufacturer’s instructions (Agilent, USA). The background-corrected intensity data were normalized by blank subtraction followed by intra-array LOWESS normalization. The peak detection was performed with Pre-defined Peak Shape detection v2.0 with *P*-value < 0.01 as the threshold of significance for a non-parametric test and a peak-score > 5 for an EVD-based score (based on T. Kaplan & N. Friedman “Model-Based Analysis of High resolution Chromatin Immunoprecipitation” Technical Report 2006–11, School of Computer Science & Engineering, Hebrew University 2006).

### Methylated DNA isolation assay-assisted CpG microarrays

Methylated DNA isolation assay (MeDIA)-assisted CpG microarray analysis was performed as described previously with slight modification [31]. Briefly, 1.0 μg sonic-fragmented genomic DNA from the test and control tissues, respectively, was incubated with 2 μg recombinant MBD2bt protein for 4 h at 4 °C on a rocking platform. The enriched, methylated DNA was amplified using a whole-genome amplification kit (GenomePlex®, Sigma, USA) according to the manufacturer’s protocol. The amplified DNA in the control and test samples was labeled with Cy5 and Cy3, respectively. The labeled samples were purified using a PCR purification kit (Qiagen, USA) and then co-hybridized to human promoter 1 M microarrays (Agilent, Santa Clara, CA, USA) according to manufacturer’s instructions.

### Microarray data analysis

The hybridized images were analyzed using an Agilent DNA Microarray Scanner (Agilent Technology, USA), and the data were quantified using the Agilent Feature Extraction software (Agilent Technology, USA). The preprocessing of raw data and the normalization steps were performed using GeneSpring 7.3.1 (Agilent, USA). The background-corrected intensity data were normalized using the intensity-dependent LOWESS method to remove the dye bias within each array as recommended by the manufacturer (Agilent, USA). To select the multiple-probes-enriched target genes, the first level of data filtering involved the removal of all probes that showed only methylation (≥ twofold enrichment of the methylated fraction in test samples versus control samples) or non-methylation (≤ twofold enrichment of the non-methylated fraction in test samples versus control samples). To increase the reliability of the results, we selected genes with probes in at least two adjacent probes.

### Chromatin immunoprecipitation assays

ChIP assays were performed, and ChIP-enriched DNA was analyzed by qPCR as described previously [32]. Briefly, cells were fixed with 1 % formaldehyde at 37 °C for 10 min, washed with cold PBS containing protease inhibitors, and lysed in Tris (pH 8.1) containing 1 % SDS, 1 mM PMSF, and complete protease-inhibitor cocktail. The cell lysates were sonicated into 500-bp chromatin fragments, diluted in ChIP dilution buffer, and immunoprecipitated overnight at 4 °C with the appropriate antibodies, with IgG control, or without antibody (no-antibody control). The next day, immune complexes were collected on ChIP-Grade Protein A/G Magnetic Beads (Thermo Fisher Scientific Inc., Rockford, IL USA), and the beads were washed to remove nonspecific binding. The DNA was eluted from the beads, the crosslinks were reversed, and the DNA was extracted. The ChIP-enriched DNA samples and the input DNA samples were analyzed by qPCR with SYBR reagent in a real-time PCR machine (ABI 7300; Applied Biosystems) using primers specific for the *TGFBI*, *Col1a1*, *CTGF*, or *PAI*-*1* promoters spanning Smad binding elements (SBEs). All reactions were performed in triplicate in a final volume of 20 μl. Dissociation curves were run to detect nonspecific amplification, and we confirmed that single products were amplified in each reaction. The qPCR data were analyzed using the 2^-ΔΔCt^ method, as described previously [32], and normalized with the input samples. The results were expressed as the fold change relative to the control. In all of the experiments, we verified that the ChIP samples obtained with specific antibodies exhibited significant enrichment relative to the IgG or no-antibody controls. The primers used for the ChIP-enriched DNA were: TGFBIp, 5′-TACCTGCCTTGAGCTCCTCC-3′ (sense) and 5′-GTGGGGTCCTCACCTTGGTA-3′ (antisense); COL1A2, 5′-CTCCGACGTGTCCCATAGTG-3′ (sense) and 5′-CTTTTGAGGCTTTCAAGGGG-3′ (antisense); PAI-1, 5′-AGTCTGGACACGTGGGGAGT-3′ (sense) and 5′-GCCAGCCACGTGATTGTCTA-3′ (antisense); and CTGF, 5′-TCTGTGAGCTGGAGTGTGCC-3′ (sense) and 5′-ACAGGGACATTCCTCGCATT-3′ (antisense).

### Western blotting

Cell lysates were fractionated by SDS-PAGE and transferred to PVDF membranes. The blocked membranes were incubated with the appropriate antibody, and the immunoreactive bands were visualized with a chemiluminescent reagent as recommended by Amersham Biosciences, Inc.

### Statistical analysis

All experiments were repeated at least three times. Data are presented as the mean ± standard error (SE), and statistical comparisons between groups were performed by one-way ANOVA followed by Tukey’s test.

## Results

### Transcription and extracellular-secretion levels of TGFBIp were high in wild-type cells compared with those in GCD2 homozygous cells and were related to H3K4me3 levels but not to DNA methylation

Not only mutation of TGFBIp gene, but also regulation of TGFBIp expression is very important cause of in GCD2.

**Fig. 1:**
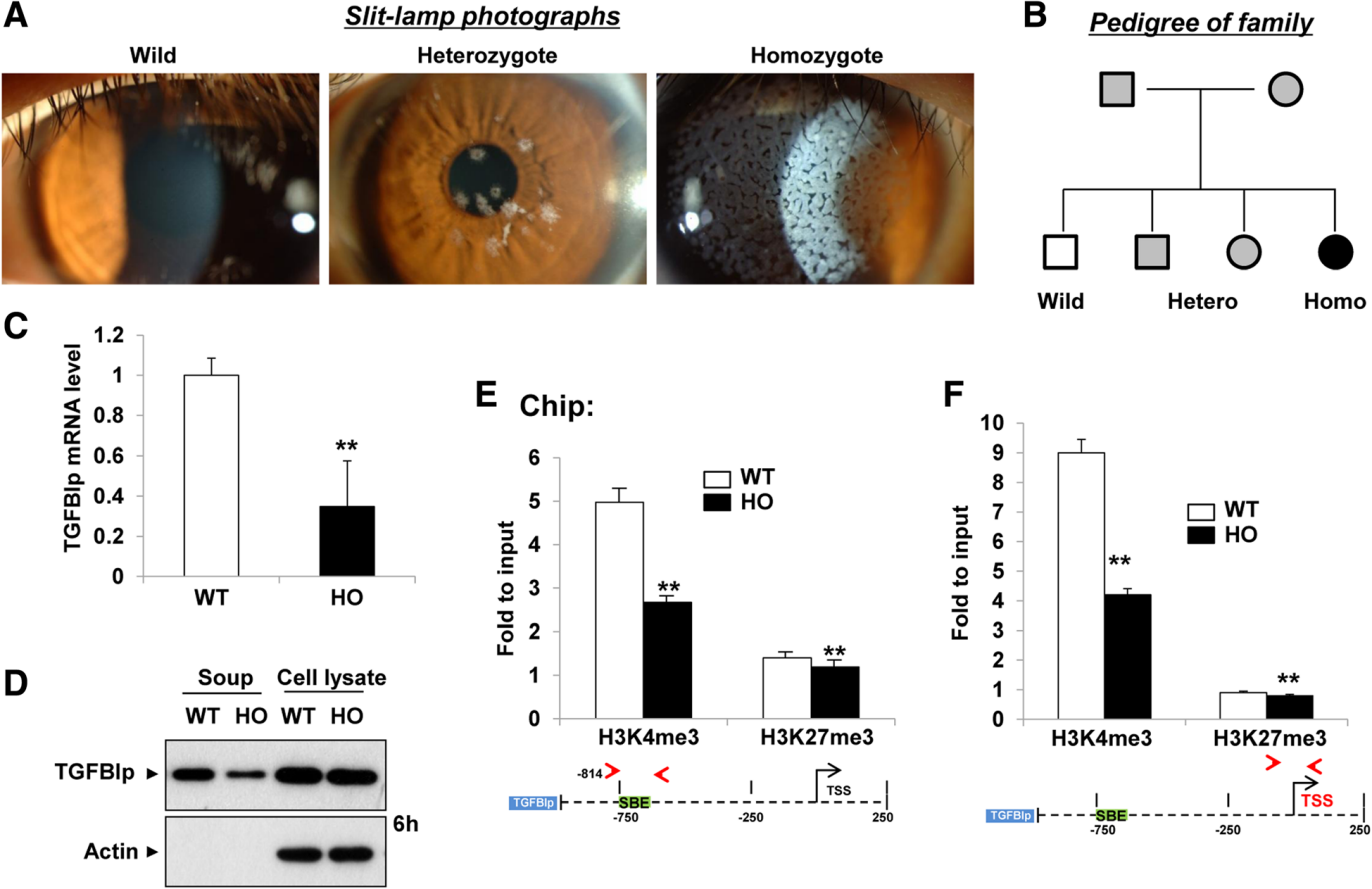
TGFBIp transcription and H3K4me3 levels in wild-type and GCD2-homozygous corneal fibroblasts. **a**, **b** Slit-lamp photographs and pedigree of wild-type, GCD2 heterozygous, and GCD2 homozygous cells. **c**, **d** The mRNA and protein levels of TGFBIp in normal and GCD2 corneal fibroblasts was determined by RT-qPCR (**c**) and western blot (**d**). Gene expression was normalized to internal control *GAPDH* gene, and results are expressed as fold stimulation over control. **e**, **f** H3K4me3 and H3K27me3 levels at the Smad binding elements on TGFBIp gene promoters and at the TSS of TGFBIp gene in wild-type and GCD2-homozygous corneal fibroblasts. ChIP assays were performed with H3K4me3 and H3K27me3 antibodies. Immunoprecipitated DNA and input DNA were subjected to qPCR with primers specific for the TGFBIp gene promoter to measure enrichment levels. qPCR data were analyzed using the 2^-ΔΔCt^ method, and data are the mean fold change relative to the input ± standard error (SE) of enrichment. (mean ± SE; **P < 0.01 vs. wild type, *n =* 3)

To investigate the mechanisms of TGFBIp gene expression, we first analyzed TGFBIp mRNA transcription levels in wild-type and GCD2-homozygous corneal fibroblasts (Fig. 1a, b). The TGFBIp mRNA transcription levels were much higher in the wild-type cells compared with those in the GCD2-homozygous cells (Fig. 1c). Moreover, the TGFBIp protein levels secreted into the culture supernatant were much higher for the wild-type cells compared with those for the GCD2-homozygous cells. The respective cell lysates from the wild-type and GCD2-homozygous cells showed similar levels of TGFBIp (Fig. 1d), however, suggesting that mutant TGFBIp accumulates in GCD2 corneal fibroblasts as a result of either impaired degradation or delayed extracellular secretion [33], causing similar intracellular TGFBIp levels in the wild-type and GCD2 homozygote cells despite the differences in TGFBIp mRNA transcription and extracellular-secretion levels.

We next examined whether the difference in TGFBIp gene expression was associated with changes in either the active epigenetic marker H3K4me3 or the repressive epigenetic marker H3K27me3 in the H3 promoter. As shown in Fig. 1e, f, the H3K4me3 levels in the Smad Binding Element (SBE) of the TGFBIp promoter and in the TSS of the TGFBIp gene were much higher in the wild-type cells compared with those in the GCD2-homozygous cells, similar to the TGFBIp mRNA transcription levels. The H3K27me3 levels were very low in both the wild-type and the GCD2-homozygous cells, however, and were not different between the two cell types. To further confirm the association between H3K4me3 levels and TGFBIp mRNA transcription levels, we analyzed wild-type (*n* = 3), GCD2-heterozygous (*n* = 1), and GCD2-homozygous (*n* = 3) corneal fibroblasts. As shown in Additional file 1: Figure S1A–C, the TGFBIp mRNA transcription levels and extracellular secreted-protein levels were much higher in the wild-type cells compared with those in the GCD2 cells and were strongly related to the H3K4me3 levels. The intracellular TGFBIp protein levels were not related to the mRNA or H3K4me3 levels in the GCD2 cells, however (Additional file 1: Figure S1D).

**Fig. 2:**
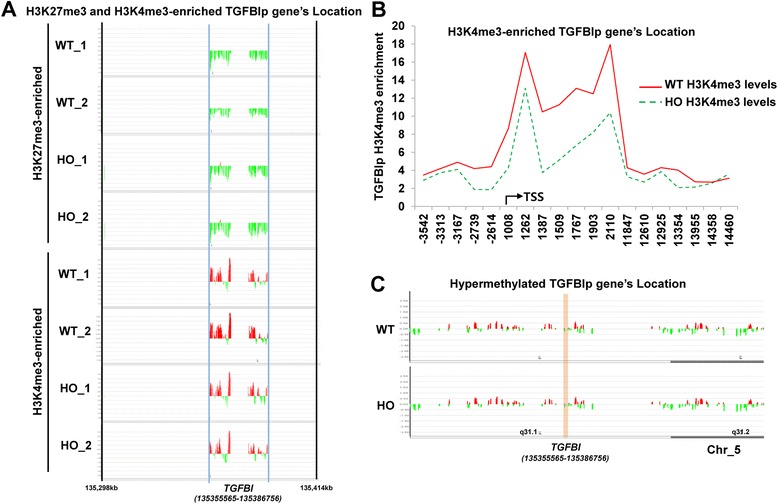
H3K4me3 and H3K27me3 levels in wild-type cells compared with those in GCD2-homozygous primary corneal fibroblasts. **a** Heat map of ChIP-chip signal for two wild-type and two GCD2-homozygous primary corneal fibroblasts. **b** Heat map of the H3K4me3-enriched TGFBIp gene location in wild-type and GCD2 primary corneal fibroblasts. Each line represents the average H3K4me3 levels in each cell type. A continuous X-axis indicates the location from the promoter region to downstream of the *TGFBI* gene. **c** Heat map of methylated DNA and CpG island regions of the TGFBIp gene in primary corneal fibroblasts

We next performed a ChIP on chip assay with H3K4me3-specific and H3K27me3-specific antibodies in wild-type (*n* = 2) and GCD2-homozygous (*n* = 2) primary corneal fibroblasts. As shown in Fig. 2a and b, the H3K4me3 levels in the TGFBIp promoter region and the downstream of transcription start site were high in the wild-type cells compared with those in the GCD2 cells, but the H3K27me3 levels were very low in both the wild-type and the GCD2 cells and were not different between the two cell types. In contrast, DNA methylation levels in the TGFBIp promoter region were very low and were not different between the two cell types (Fig. 2c). Collectively, these results suggest that the mRNA transcription and extracellular-secretion levels of TGFBIp were high in the wild-type cells compared with those in the GCD2-homozygous cells and were related to the H3K4me3 levels but not to DNA methylation over the TGFBI locus.

### TGFβ1 increased the expression of TGFBIp and ECM-associated genes and Smad3 recruitment to the TGFBIp and ECM-associated gene promoters

**Fig. 3:**
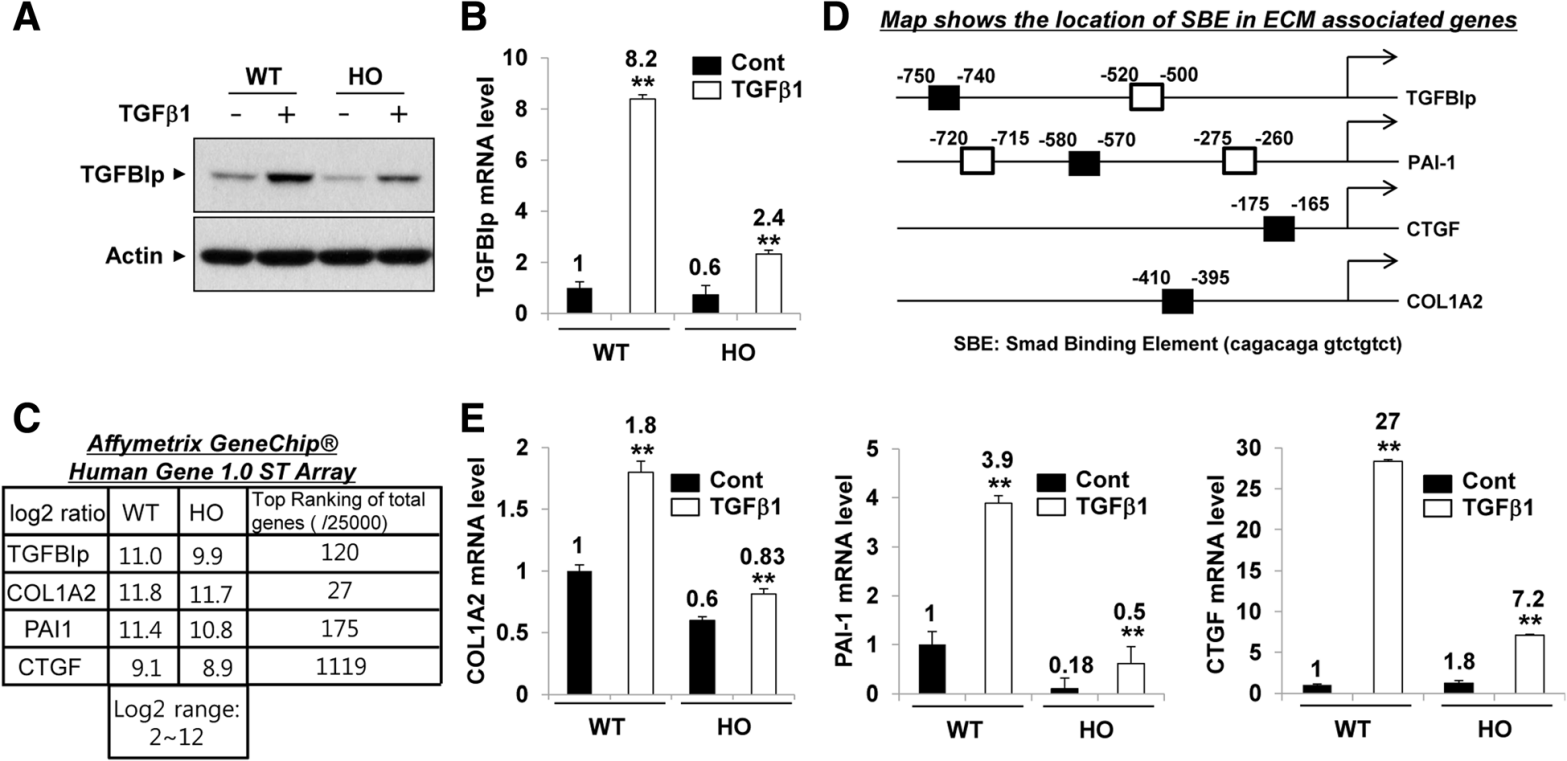
TGFβ1-induced expression of TGFBIp and ECM-associated genes in wild-type and GCD2-homozygous corneal fibroblasts. **a**, **b** Serum-depleted wild-type and GCD2 corneal fibroblasts were stimulated with TGFβ1 (5 ng/ml) for 8 h, and protein and mRNA levels of TGFBIp were analyzed by western blot (**a**) and RT-qPCR (**b**). **b** Gene expression was normalized to that of GAPDH (internal control). Results are expressed as fold stimulation relative to the wild-type control (mean ± standard error (SE); **P < 0.01 vs. wild type, *n =* 3). **c** Total mRNA was isolated from wild-type and GCD2-homozygous corneal fibroblasts, and gene-expression profiles were assessed using an Affymetrix gene chip. Expression of TGFBIp and ECM-associated genes (COL1A2, CTGF, and PAI-1) is presented as the log2 ratio. **d** Map shows the locations of Smad binding elements (SBEs) and the primers used for ChIP-qPCR of the TGFBIp, COL1A21, CTGF, and PAI-1 gene promoters. Open and filled boxes are Smad binding elements. Filled boxes are real engaged positions of Smad in corneal fibroblast. **e** Serum-depleted wild-type and GCD2-homozygous corneal fibroblasts were stimulated with TGFβ1 (5 ng/ml) for 8 h, and mRNA levels of ECM-associated genes (COL1A2, CTGF, and PAI-1) were analyzed by RT-qPCR. Gene expression was normalized to that of GAPDH (internal control), and results are expressed as fold stimulation over the wild-type control (mean ± standard error (SE); **P < 0.01 vs. wild type, *n =* 3)

We examined whether TGFBIp expression could be induced by TGFβ1 treatment in corneal fibroblasts. As shown in Fig. 3a and b, TGFBIp protein and mRNA levels were significantly increased by TGFβ1 in the wild-type cells, and the GCD2-homozygous cells showed much lower TGFBIp levels compared with the wild-type cells. Furthermore, As shown in Fig. 3c, the ECM-associated genes for COL1A2, CTGF, and PAI-1 were highly expressed together with TGFBIp in both the wild-type and the GCD2 corneal fibroblasts. The promoters of those ECM-associated genes contain the SBE, which is a TGFβ1 downstream effector-signaling molecule (Fig. 3d). Therefore, we examined whether the ECM-associated gene expression could be induced by TGFβ1 treatment in corneal fibroblasts. As shown in Fig. 3e, the COL1A2, PAI-1, and CTGF mRNA levels were significantly increased by TGFβ1 in wild-type corneal fibroblasts, whereas the GCD2 homozygous cells showed much smaller increases in ECM-associated gene expression levels under same conditions (Fig. 3e).

**Fig. 4:**
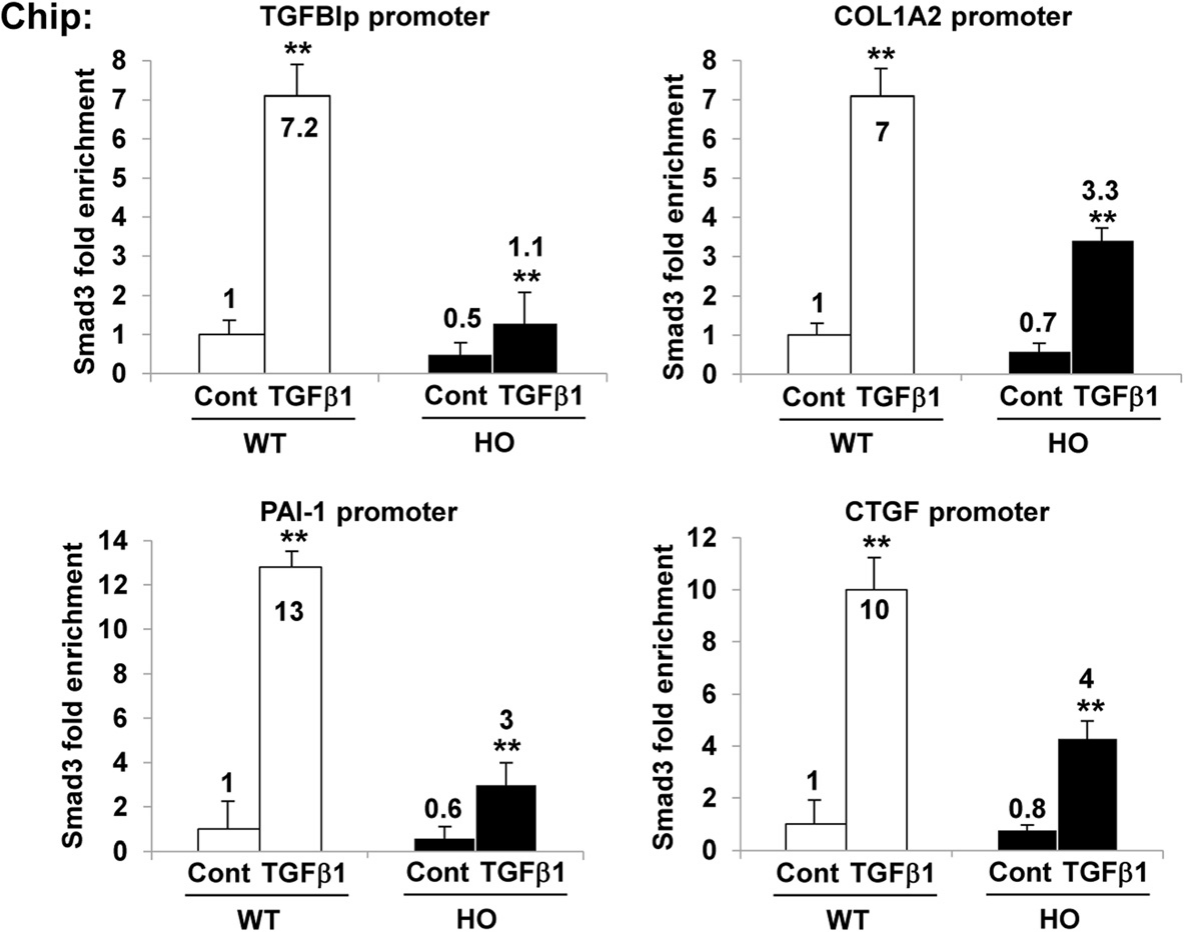
TGFβ1 enhanced Smad3 recruitment to the TGFBIp and ECM-associated gene promoters. Smad3 recruitment at the indicated gene promoters in wild-type and GCD2 homozygous corneal fibroblasts was stimulated by TGFβ1 (5 ng/ml) for 8 h. Smad3 recruitment was determined by ChIP assays with Smad3 antibody. Results were normalized to the input, and Smad3 occupancy was expressed as the fold enrichment over respective wild-type control samples (mean ± standard error (SE); **P < 0.01 vs. wild type, *n =* 3)

Next, we examined whether TGFβ1 altered the Smad3 occupancy on the promoters of the TGFBIp, COL1A2, PAI-1, and CTGF genes using ChIP assays with Smad3 antibodies. As shown in Fig. 4, Smad3 recruitment was significantly increased on the TGFBIp, COL1A2, PAI-1, and CTGF promoters after TGFβ1 stimulation in the GCD2-homozygous cells relative to that in the wild-type cells (Fig. 4). The GCD2-homozygous cells showed very small increases in Smad3 recruitment to the TGFBIp and ECM-associated gene promoters, however (Fig. 4), suggesting that TGFβ1 increases the expression of TGFBIp and ECM-associated genes by increasing Smad3 recruitment to the TGFBIp and ECM-associated gene promoters in corneal fibroblasts.

### TGFβ1 enhanced H3K4me1/3 levels on the promoters of TGFBIp and ECM-associated genes

**Fig. 5:**
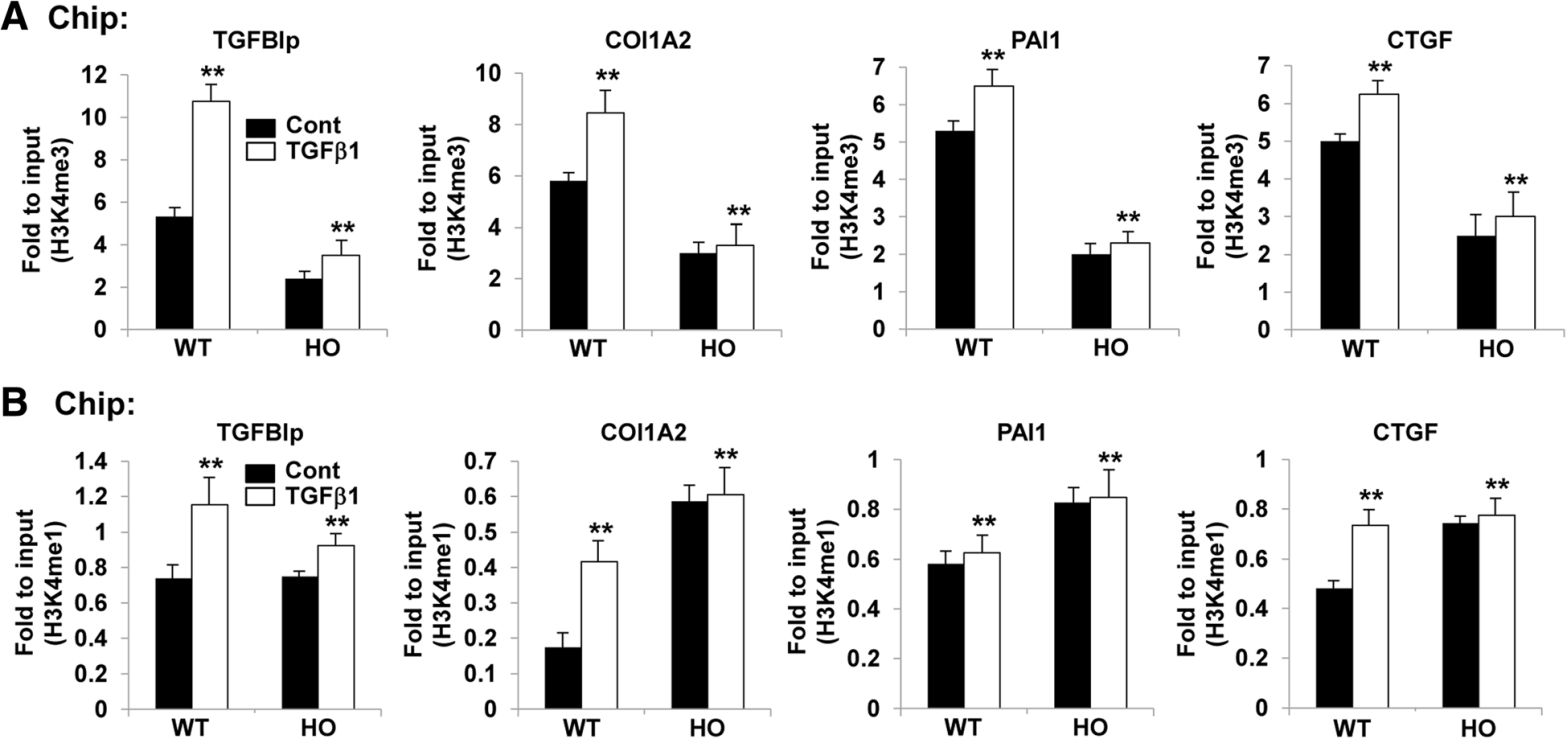
TGFβ1 increased H3K4me1/3 levels on TGFBIp and ECM-associated gene promoters in wild-type and GCD2-homozygous cells. **a**, **b** Bar graphs showing H3K4me3 (**a**) and H3K4me1 (**b**) levels at the indicated gene promoters in control and TGFβ1 (5 ng/ml)-stimulated wild-type and GCD2-homozygous corneal fibroblasts. ChIP assays were performed with H3K4me3 and H3K4me1 antibodies. Immunoprecipitated DNA and input DNA were subjected to qPCR with primers specific for the indicated gene promoters to measure enrichment levels. qPCR data were analyzed using the 2^-ΔΔCt^ method, and data are shown as the mean fold change of the input ± standard error (SE) of enrichment. (mean ± SE; **P < 0.01 vs. wild type, *n =* 3)

To investigate whether TGFβ1 could alter promoter levels of H3K4me, we performed ChIP assays with H3K4me1 or H3K4me3 antibodies. As shown in Fig. 5a and b, TGFβ1 strongly increased H3K4me1 and H3K4me3 levels on the TGFBIp and ECM-associated gene promoters in the wild-type corneal fibroblasts. But only weakly increased those levels in the GCD2-homozygous cells under the same conditions (Fig. 5a, b). The increases in promoter H3K4me1/3 levels were correlated with the increased expression of the associated genes induced by TGFβ1. In contrast, levels of the repressive epigenetic marker H3K27me3 decreased in TGFβ1-stimulated wild-type corneal fibroblasts (Additional file 1: Figure S2A), but there was no significant effect of TGFβ1 on H3K27me3 levels in the GCD2-homozygous cells (Additional file 1: Figure S2A). Furthermore, levels of H3K9me3, another repressive epigenetic marker, were not significantly changed in either the wild-type or the GCD2-homozygous cells under same conditions (Additional file 1: Figure S2B), suggesting that increases in H3K4me1/3 and decreases in H3K27me3 on the target-gene promoters may be involved in the TGFβ1-induced up-regulation of TGFBIp and ECM-associated genes in corneal fibroblasts.

### TGFβ1 increased MLL1 recruitment to the TGFBIp and ECM-associated gene promoters

**Fig. 6:**
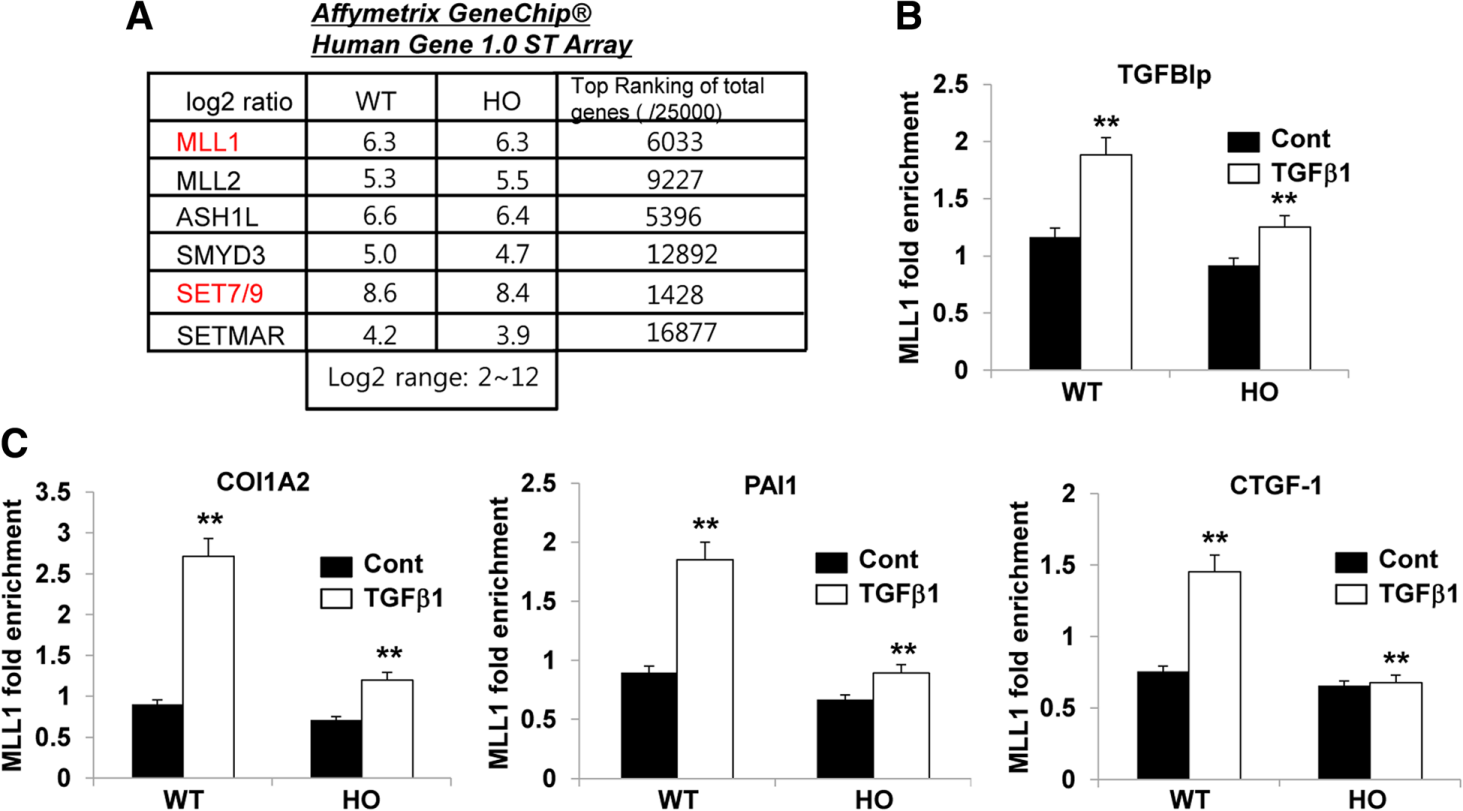
TGFβ1 enhanced MLL1 recruitment on TGFBIp and ECM-associated gene promoters. **a** Total mRNA was isolated from wild-type and GCD2-homozygous corneal fibroblasts, and gene-expression profiles were assessed using an Affymetrix gene chip. The expression levels of H3K4 methyltransferase genes are presented as the log2 ratio. **b**, **c** MLL1 recruitment to the indicated gene promoters in wild-type and GCD2 corneal fibroblasts stimulated by TGFβ1 (5 ng/ml) for 8 h. MLL1 recruitment was determined by ChIP assays with MLL1 antibody. Results were normalized to the input, and MLL1 occupancy was expressed as the fold enrichment over the respective wild-type control samples (mean ± standard error (SE); **P < 0.01 vs. wild type, *n =* 3)

To identify genes that regulate the H3K4me1/3 induced by TGFβ1, we analyzed the expression of H3K4-methyltransferase genes in wild-type and and GCD2 corneal fibroblasts by microarray analysis. As shown in Fig. 6a, MLL1, an H3K4 tri-methyltransferase, and SET7/9, an H3K4 mono- methyltransferase, were expressed in wild-type and GCD2 cells. Next, we examined whether TGFβ1 alters the MLL1 occupancy on the promoters of the TGFBIp, COL1A2, PAI-1, and CTGF genes using ChIP assays with MLL1 antibodies. As shown in Fig. 6b and c, MLL1 recruitment was significantly increased in the TGFBIp, COL1A2, PAI-1, and CTGF promoters after TGFβ1 stimulation in the wild-type corneal fibroblasts (Fig. 6b, c). The GCD2 homozygous cells showed very weak increased recruitment of MLL1 to the TGFBIp and ECM-associated gene promoters (Fig. 6b, c). The MLL1 recruitment pattern was quite similar to the pattern of increased H3K4me3 (Fig. 5a), suggesting a key role for MLL1 in the TGFβ1-mediated increase in H3K4me3 during the induction of the TGFBIp and ECM genes.

### MLL1 and SET7/9 knockdown attenuated the TGFβ1-induced expression of the TGFBIp and ECM-associated genes

**Fig. 7:**
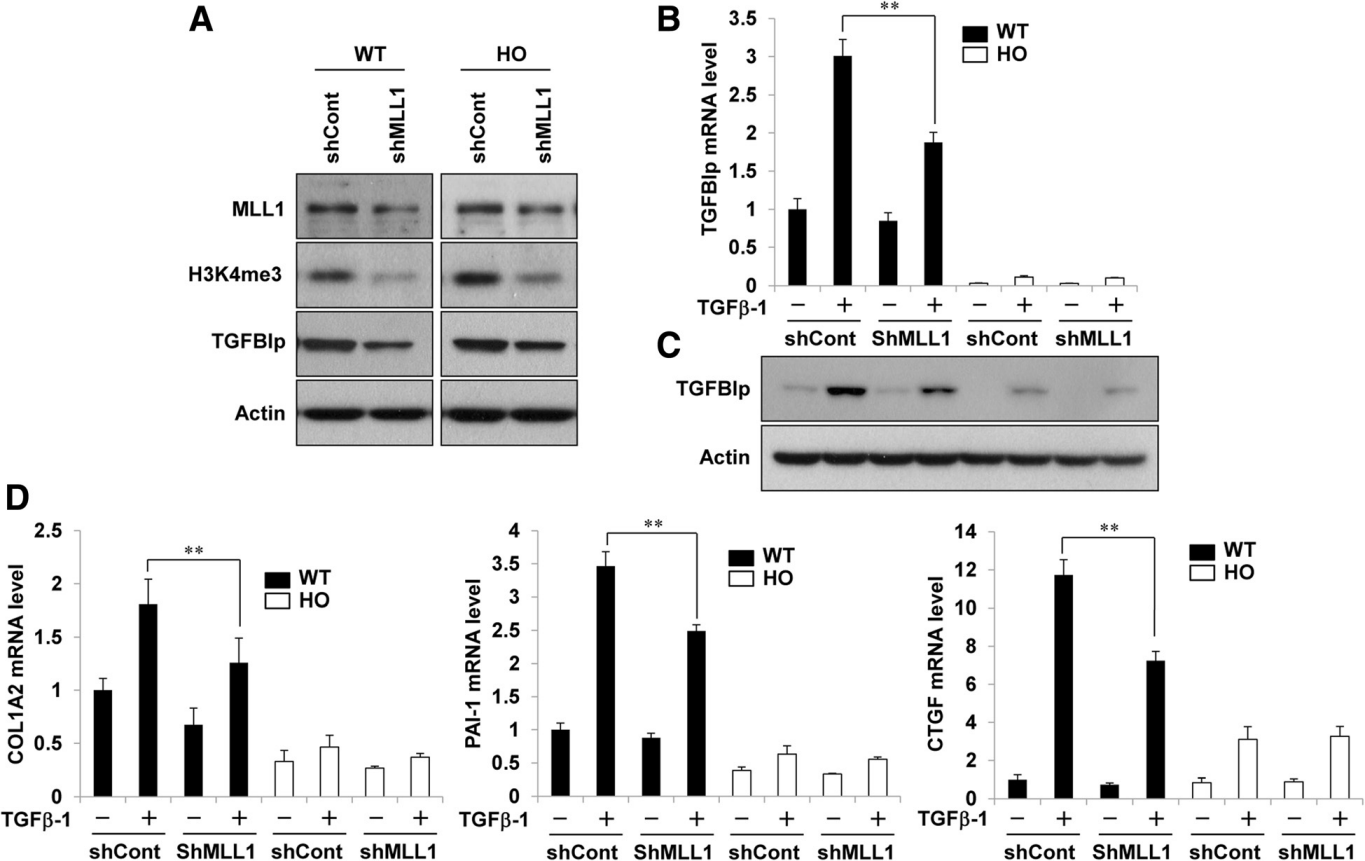
MLL1 knockdown attenuated the TGFβ1-induced expression of TGFBIp and ECM-associated genes. **a** Wild-type and GCD2-homozygous corneal fibroblasts were infected with MLL1 or control shRNA lentivirus. After puromycin selection, MLL1, H3K4me3, TGFBIp, and actin protein levels were analyzed by western blot. **b**-**d** Wild-type and GCD2-homozygous corneal fibroblasts were infected with MLL1 or control shRNA lentivirus. After puromycin selection, infected cells were stimulated with TGFβ1 (5 ng/ml) for 8 h, and mRNA and protein levels of TGFBIp were analyzed by RT-qPCR (**b**) and western blot (**c**). mRNA levels of ECM-associated genes were analyzed by RT-qPCR (**d**). Gene expression was normalized to that of GAPDH (internal control), and results are expressed as fold stimulation over control shRNA-infected wild-type control (mean ± standard error (SE); **P < 0.01 vs. control, *n =* 3)

Corneal fibroblasts were first infected with lentivirus containing shRNA targeting MLL1 (shMLL1) or control shRNA (shCont), and protein levels were analyzed by western blot. As shown in Fig. 7a, MLL1 protein levels were significantly reduced in the wild-type and GCD2 cells infected with shMLL1 lentivirus compared with those in wild-type and GCD2 cells infected with shCont lentivirus. We next examined whether MLL1 shRNA can affect H3K4me3 in corneal fibroblasts, because previous studies showed that MLL1 regulates H3K4me3 in embryonic fibroblasts and immune cells [34–36]. Global H3K4me3 levels were significantly reduced in both the wild-type cells and the GCD2 cells infected with shMLL1 lentivirus (Fig. 7a). Also, TGFBIp protein levels were reduced in cells infected with shMLL1 lentivirus. Furthermore, shMLL1 significantly attenuated TGFβ1-induced TGFBIp protein and mRNA levels in wild-type corneal fibroblasts compared with shCont (Fig. 7b, c). The GCD2-homozygous cells showed very weak TGFBIp expression and inhibition induced by TGFβ1 and shMLL1, respectively (Fig. 7b, c). TGFβ1-induced COL1A2, PAI-1, and CTGF mRNA levels were inhibited by shMLL1 (Fig. 7d), whereas no significant increase and decrease in response to TGFβ1 and shMLL1 were observed in the GCD2-homozygous cells (Fig. 7d), suggesting a key role for MLL1 in modulating TGFβ1 responses in corneal fibroblasts.

To investigate the role of SET7/9 in the expression of the TGFBIp and ECM genes, cells were infected with shSET7/9 or shCont lentivirus, and protein levels were analyzed by western blot. As shown in Additional file 1: Figure S3A, total H3K4me1 levels were clearly decreased in the SET7/9 knockdown cells compared with the shCont cells. In addition, the TGFBIp protein levels were inhibited in the cells infected with shSET7/9 (Additional file 1: Figure S3A). Furthermore, TGFβ1-induced TGFBIp protein and mRNA levels were inhibited by shSET7/9 (Additional file 1: Figure S3B, C) in wild-type corneal fibroblasts. The GCD2-homozygote cells showed very weak TGFBIp expression and inhibition induced by TGFβ1 and shSET7/9, respectively (Additional file 1: Figure S3B, C). TGFβ1-induced COL1A2, PAI-1, and CTGF mRNA levels were also partially inhibited by shSET7/9 lentivirus infection (Additional file 1: Figure S3D), whereas no significant increase and decrease induced by TGFβ1 and shSET7/9, respectively, was observed in the GCD2-homozygous cells (Additional file 1: Figure S3D). Thus, SET7/9 likely partially mediates the induction of H3K4me1 by TGFβ1 in corneal fibroblasts. Collectively, these results suggest that MLL1 and SET7/9 are involved in basal TGFBIp expression and TGFβ1-induced TGFBIp and ECM-associated gene expression.

### MLL1 and SET7/9 knockdown attenuated the TGFβ1-induced increase in H3K4me1/3 levels on the promoters of the TGFBIp and ECM-associated genes

**Fig. 8:**
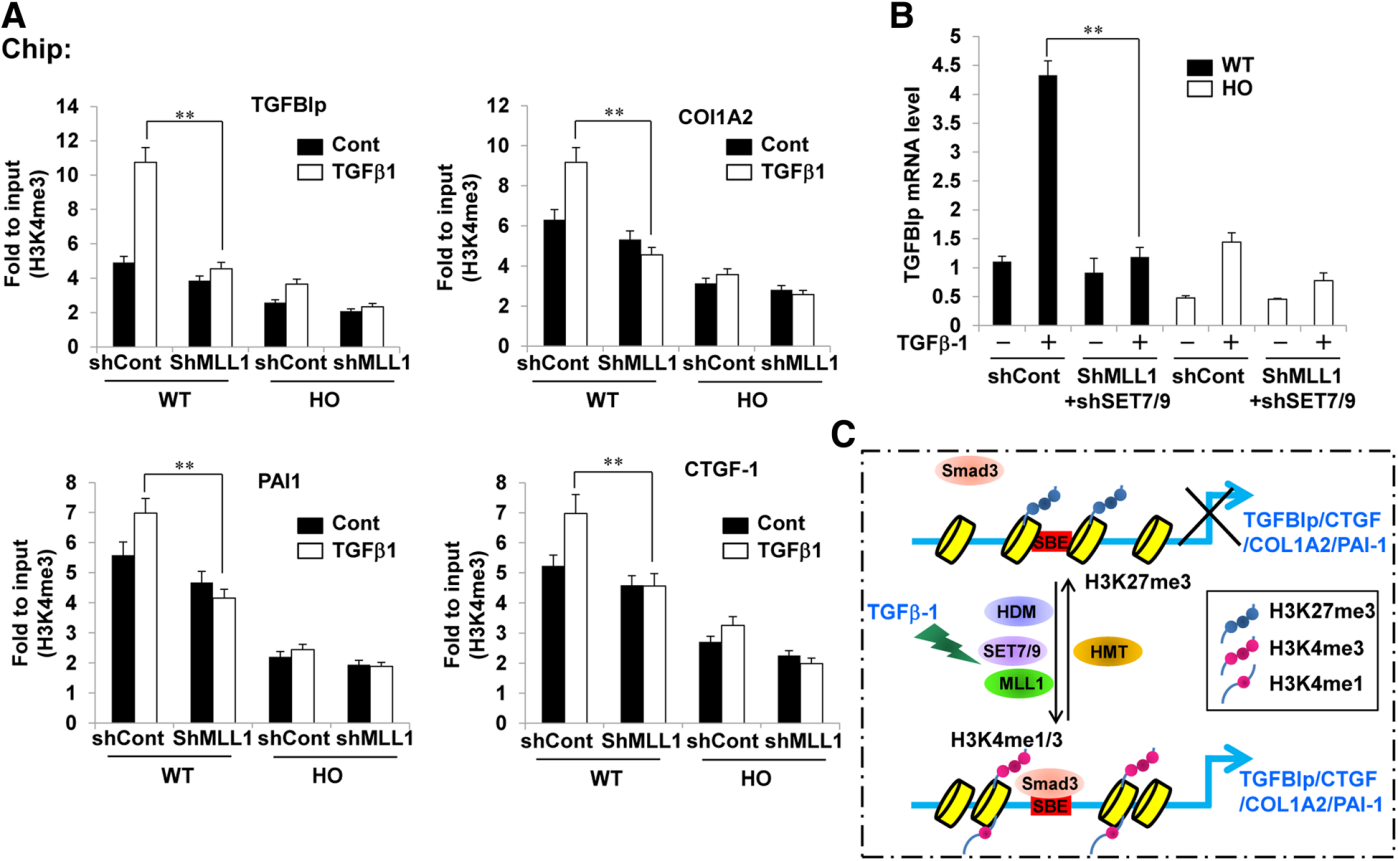
MLL1 knockdown attenuated TGFβ1-induced increases in H3K4me3 on the promoters of TGFBIp and ECM-associated genes. **a** Wild-type and GCD2-homozygous corneal fibroblasts were infected with MLL1 or control shRNA lentivirus. After puromycin selection, infected cells were stimulated with TGF-β1 (5 ng/ml) for 8 h, and H3K4me3 levels at the indicated gene promoters were analyzed. ChIP assays were performed with H3K4me3 antibody. Immunoprecipitated DNA and input DNA were subjected to qPCR with primers specific for the indicated gene promoters to measure enrichment levels. qPCR data were analyzed using the 2^-ΔΔCt^ method, and data are presented as mean fold change of the input ± standard error (SE) of enrichment. (mean ± SE; **P < 0.01 vs. control, *n =* 3). **b** Wild-type and GCD2-homozygous corneal fibroblasts were double infected with MLL1 and SET7/9 or control shRNA lentivirus. After puromycin selection, infected cells were stimulated with TGFβ1 (5 ng/ml) for 8 h, and mRNA levels of TGFBIp were analyzed by RT-qPCR. Gene expression was normalized to that of GAPDH (internal control), and results are expressed as fold stimulation over control shRNA-infected wild-type control (mean ± standard error (SE); **P < 0.01 vs. control, *n =* 3) (**c**) A model showing that in corneal fibroblasts reaching the quiescent state, transcriptional repression of TGFBIp and ECM-associated genes occurs through heterochromatin formation under no stimulation. Lysine 27 from histone H3 (H3K27) near Smad binding elements (SBEs) within promoters was tri-methylated. During TGFβ1 stimulation, MLL1 and SET7/9 were activated, and their recruitment to SBEs was increased and recruitment of Smad to SBEs was increased. H3K4me1/3 became more methylated, and H3K27me3 was de-methylated on the gene promoters, creating a favorable environment for Smad3 binding

We tested whether the knockdown of MLL1 and SET7/9 can decrease active H3K4me1/3 markers on the promoters of the TGFBIp and ECM-associated genes. The knockdown of MLL1 and SET7/9 significantly decreased TGFβ1-mediated H3K4me3 (Fig. 8a) and H3K4me1 (Additional file 1: Figure S4) levels on the TGFBIp, COL1A2, PAI-1, and CTGF promoters. These findings show the mediatory roles of MLL1 and SET7/9 in TGFβ1-induced epigenetic events on the promoters of the TGFBIp and ECM genes and in the subsequent expression of those genes.

To better understand the roles of MLL1 and SET7/9 in TGFBIp expression, cells were co-infected with shMLL1 and shSET7/9, and TGFBIp mRNA levels were analyzed by RT-qPCR. As shown in Fig. 8b, shMLL1 and shSET7/9 co-infection completely inhibited TGFβ1-induced TGFBIp mRNA levels compared with shMLL1 or shSET7/9 single infection, providing evidence that both MLL1 and SET7/9 mediate TGFβ1-induced TGFBIp expression by regulating H3K4me1/3.

## Discussion

In GCD2 pathogenesis, mutations in the *TGFBI* gene important, but the regulation of TGFBIp expression is also very important. We confirmed that mRNA transcription and extracellular-secretion levels of TGFBIp were high in wild-type corneal fibroblasts compared with those in GCD2-homozygous cells. The expression pattern was strongly related to H3K4me3 levels but not to DNA methylation over the TGFBI locus. Intracellular TGFBIp levels were similar in wild-type and GCD2-homozygous cells, although TGFBIp mRNA transcription and extracellular secreted-protein levels were much lower in GCD2-homozygous cells compared with wild-type cells, because mutant TGFBIp can accumulate in GCD2 corneal fibroblasts as a result of either impaired degradation or delayed extracellular secretion [33]. The abnormal conditions within GCD2 cells caused by mutant-TGFBIp accumulation may weaken responses to exogenous stimuli such as TGFβ1 compared with those in wild-type cells.

Alternately, the observed differences in histone modification in GCD2 fibroblasts compared to normal cells may be due to the reduced expression of TGFBI in the mutant cells. Much less TGFBIp is secreted from mutant than from wild type cells. However, intracellular TGFBIp is at similar levels in both cell types. Given the reduced secretion of the protein in mutant cells, less transcription is required to maintain intracellular protein levels and negative feedback would suppress transcription in mutant relative to wild type cells. Thus, it is expected that levels of H3K4me3, a well-known marker of active transcription, would be reduced at the promoter of the *TGFBI* gene. Our data show that the transcription of *TGFBI* and associated ECM genes is increased upon treatment with TGFβ1 and that H3K4me3 levels again correlate with expression. These effects are reduced, but not eliminated, in mutant cells. The association of active histone modifications with levels of transcription may not represent proof of a causative link. Rather, it is more likely that the accumulation of TGFBIp is driving the altered transcription pattern in mutant cells.

In our study, we found that TGFβ1 can up-regulate TGFBIp and ECM-associated genes such as COL1A2, PAI-1, and CTGF in corneal fibroblasts. TGFBIp expression induced by TGFβ1 was seen previously in corneal fibroblasts, but our study is the first to demonstrate increases in the expression of other ECM genes that may coagulate with TGFBIp in the corneas of patients with GCD2. We observed that TGFβ1 increased Smad3 recruitment to the TGFBIp and ECM-associated gene promoters in corneal fibroblasts. TGFBIp and ECM-associated genes have conserved SBEs in their promoters, suggesting that gene expression could be similarly regulated under conditions such as TGFβ1 stimulation.

We examined changes in key epigenetic chromatin markers, including H3K27me3 and H3K4me1/3 levels, on the TGFBIp and ECM-associated gene promoters. Our results showed that those markers, along with H3K27 histone de-methylases (HDMs) and the MLL1 and SET7/9 HMTs, are involved in the TGFβ1-induced up-regulation of TGFBIp and ECM-associated genes in corneal fibroblasts. We showed that the up-regulation of H3K4me1/3 markers usually associated with active chromatin occurs in parallel with the down-regulation of H3K27me3 by TGFβ1, suggesting that those regulatory mechanisms can further contribute to the increased gene expression. Recent studies showed that methylated H3K4 correlates with transcriptionally competent chromatin and is associated with active genes [25–27], supporting our observation that TGFβ1 increased the expression of the TGFBIp and ECM genes in corneal fibroblasts and that such expression was positively correlated with increased H3K4me1and H3K4me3 levels on the associated promoters.

Increasing evidence shows that H3K27me3 markers are recognized by polycomb protein and generally correlate with gene silencing and transcriptional repression [26, 37, 38]. Our results showed for the first time that TGFβ1 decreased H3K27me3 levels on the TGFBIp and ECM gene promoters and that those levels were inversely correlated with the increased expression of the genes, further supporting the notion that a relief of transcriptional repression caused by decreases in repressive chromatin histone modifications may contribute to the increased expression of TGFBIp and ECM genes induced by TGFβ1. Collectively, our observations show that increases in H3K4me1/3 and decreases in H3K27me3 at the TGFBIp and ECM gene promoters in the presence of TGFβ1 in corneal fibroblasts are correlated with the TGFβ1-induced up-regulation of those genes.

Furthermore, we showed not only an increase in the recruitment of the H4K4 HMTs MLL1 and SET7/9 at the TGFBIp and ECM gene promoters but also a decrease in the recruitment of H4K27 HMT in corneal fibroblasts stimulated by TGFβ1, suggesting that the H4K4 HMT MLL1 is involved in the TGFβ1-induced up-regulation of the TGFBIp and ECM genes in corneal fibroblasts. This was supported by our observations that MLL1 and SET7/9 gene silencing partially, but significantly, blocked the expression of the TGFβ1-induced TGFBIp and ECM genes. The knockdown of MLL1 and SET7/9 with shRNAs could decrease global H3K4me1 and H3K4me3 levels, suggesting that H3K4me1/3 mediated by MLL1 and SET7/9 plays a key role in TGFBIp and ECM-associated gene expression and that MLL1 and SET7/9 are potential therapeutic targets for the treatment of ECM-accumulation disorders such as GCD2. The observed increases in H3K4me3 might synergize with other complementary events occurring on the TGFBIp and ECM-associated gene promoters, such as increases in H3K4me1 and decreases in H3K27me3, to enhance TGFBIp and ECM gene expression in response to TGFβ1. In addition, the HMTs and HDMs, as well as the HATs and histone deacetylases, regulating the complementary chromatin markers may also play cooperative roles. Additional studies are needed to assess those factors, including the role of the HMTs that mediate H3K4me1/3 and H3K27me3. It was suggested that the H3K4me1/3 mediated by MLL1 and SET7/9 functions in transcriptional activation by competing with histone deacetylases to enhance H3-K9 acetylation and prevent H3-K9 methylation [39, 40]. MLL1 and SET7/9 can methylate non-histone proteins including p53 [41, 42], DNMT1 [43], TAF10 [44], and p65 [45, 46]. Furthermore, MLL1 and SET7/9 could regulate a subset of TNFα-induced, NF-κB-dependent inflammatory genes in monocytes [47] and the high glucose-induced expression of NF-κB p65 and inflammatory genes in endothelial cells [48], indicating the diverse physiologic roles of MLL1 and SET7/9 in gene transactivation. Similarly, MLL1 and SET7/9 may also methylate other non-histone proteins and possibly even Smads in corneal fibroblasts. Further studies are needed to evaluate those possibilities. In the future, analogous approaches should be explored to examine epigenetic mechanisms of regulating the expression of other matrix genes in corneal epithelial cells exposed to TGFβ1. Epigenetic mechanisms may affect ECM expression by regulating the expression of metalloproteases and other proteases, because histone modification has been shown to regulate metalloproteases in some cells [49]. Taken together, our results show that TGFβ1 can promote significant changes in H3K4 and H3K27 methylation on gene promoters in corneal fibroblasts that correlate with parallel increases in the expression of genes related to ECM accumulation and GCD2 pathogenesis.

## Conclusions

In summary, our findings clearly show that epigenetic histone methylation modulates the expression of TGFBIp and ECM genes in corneal fibroblasts. In corneal fibroblasts reaching the quiescent state, the transcriptional repression of TGFBIp and ECM-associated genes occurs through heterochromatin formation with no external stimulation. H3K27 residues near SBEs within promoters are tri-methylated. During TGFβ1 stimulation, MLL1 and SET7/9 are activated, increasing their recruitment to SBEs. H3K4me1/3 becomes more methylated, and H3K27me3 becomes de-methylated on gene promoters, creating a favorable environment for Smad3 binding, resulting in the increased expression of TGFBIp and ECM-associated genes (Fig. 8c). The effective epigenetic regulation of histone H3K4me during the TGFβ1-mediated expression of TGFBIp and ECM genes in corneal fibroblasts could be used to create cornea-protective therapies for granular corneal dystrophy.

## Additional file

Additional file 1: Figure S1. TGFBIp transcription was high in wild-type cells compared with that in GCD2 corneal fibroblasts and was related to H3K4me3 levels. (A) H3K4me3 levels at the Smad binding elements of TGFBIp gene promoters in wild-type, GCD2-heterozygous, and GCD2-homozygous corneal fibroblasts. ChIP assays were performed with H3K4me3 antibody. (mean ±SE). (B) mRNA levels of TGFBIp in corneal fibroblasts were determined by RT-qPCR. (C, D) Protein levels of TGFBIp in cell lysates or culture supernatants were determined by western blot. **Figure S2.** TGFb1 had a minor effect on H3K27me3 and H3K9me3 modification on the TGFBIp and ECM-associated gene promoters. Bar graphs showing H3K27me3 (A) and H3K9me3 (B) levels on the indicated gene promoters in control and TGFb1 (5 ng/ml)-stimulated corneal fibroblasts. ChIP assays were performed with H3K27me3 and H3K9me3 antibodies. (mean ±SE; **P < 0.01 vs. control, n = 3). **Figure S3.** SET7/9 knockdown attenuated the TGFb1-induced expression of TGFBIp and ECM-associated genes. Wild-type and GCD2-homozygous corneal fibroblasts were infected with SET7/9 or control shRNA lentivirus. After puromycin selection, SET7/9, H3K4me1, TGFBIp, and actin protein levels were analyzed by western blot (A) and infected cells were stimulated with TGFb1 (5 ng/ml) for 8 h, and mRNA and protein levels of TGFBIp were analyzed by RT-qPCR (B) and western blot (C). mRNA levels of ECM associated genes were analyzed by RT-qPCR (D). (mean ±standard error (SE); **P < 0.01 vs. control, n = 3). **Figure S4.** SET7/9 knockdown attenuated TGFb1-induced increases in H3K4me1 levels at the promoters of TGFBIp and ECM-associated genes. SET7/9 or control shRNA lentivirus infected cells were stimulated with TGFb1 (5 ng/ml) for 8 h, and H3K4me1 levels at the indicated gene promoters were analyzed. ChIP assays were performed with H3K4me1 antibody. (mean ± SE); **P < 0.01 vs. control, n = 3).
