# Supplementary figures and images for: Histone methylation levels correlate with TGFBIp and extracellular matrix gene expression in normal and granular corneal dystrophy type 2 corneal fibroblasts
Source: BMC Med Genomics. 2015 Nov 9;8:74. doi: 10.1186/s12920-015-0151-8 (PMC4638082; doi:10.1186/s12920-015-0151-8)

# Supplementary Fig. 1

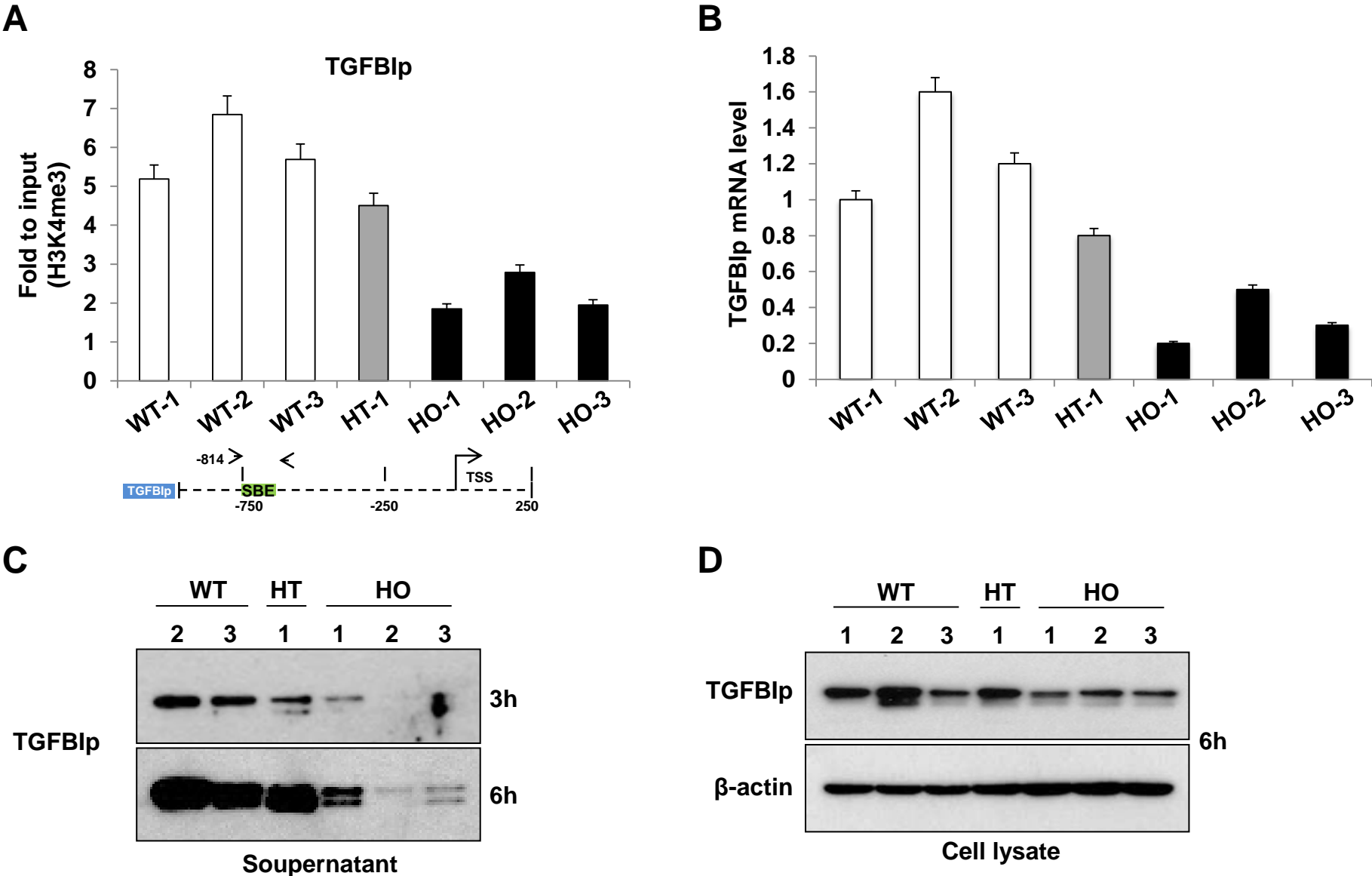

# Supplementary Fig. 2

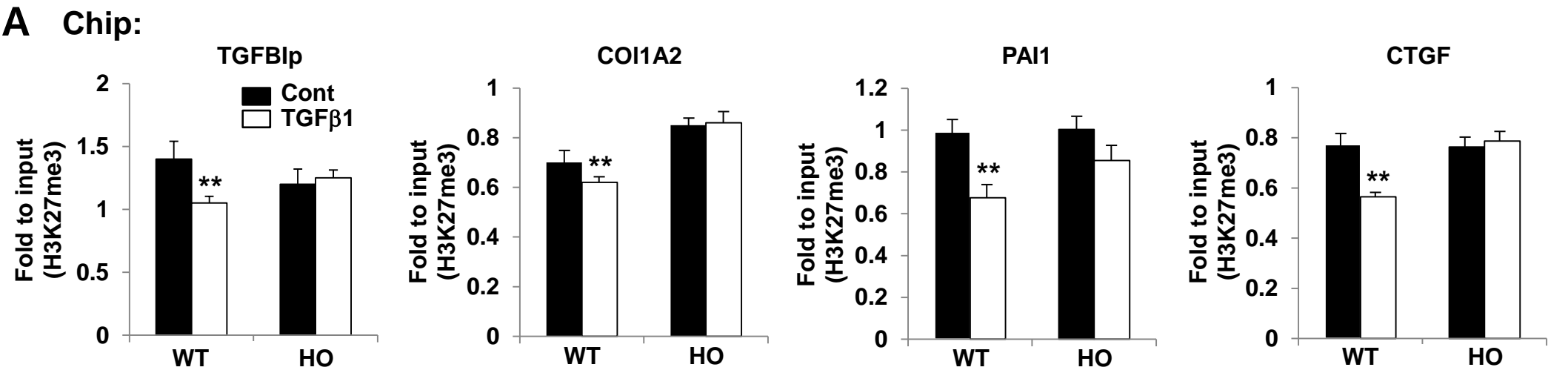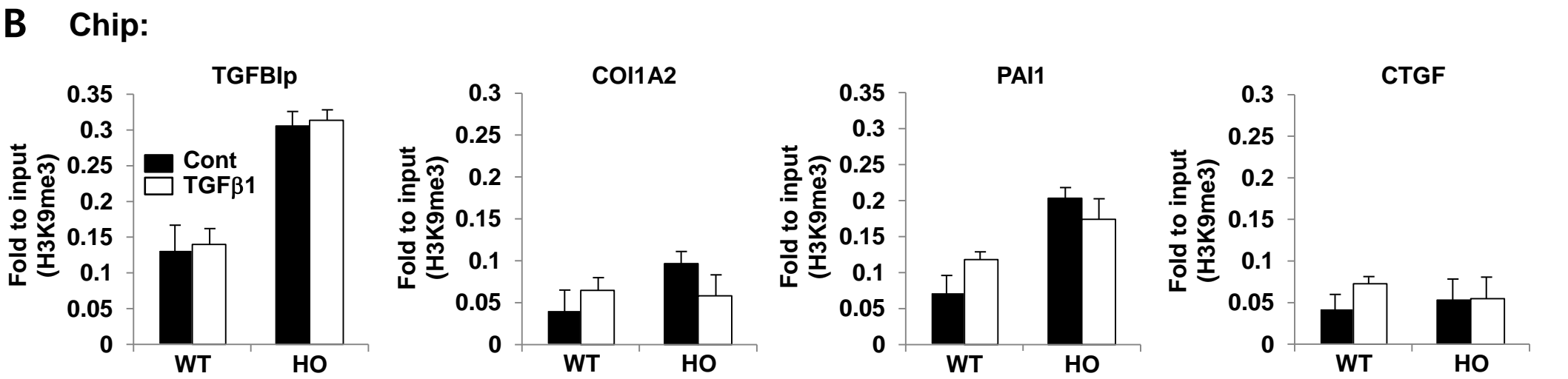

Supplementary Fig. 3

A

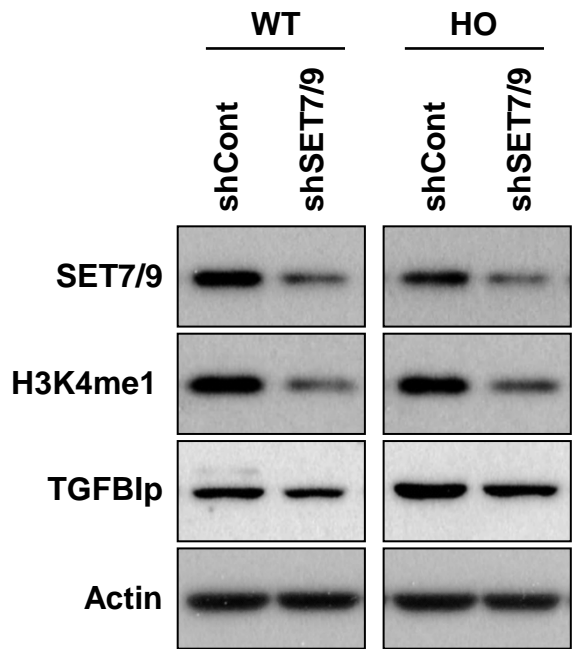

B

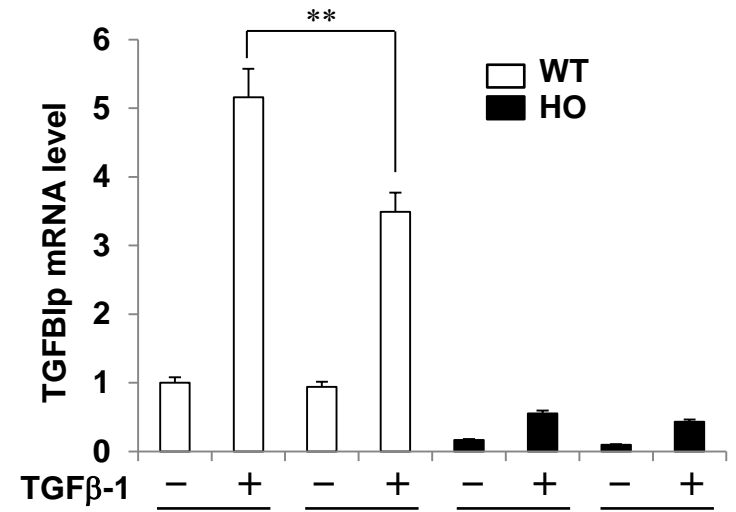

C

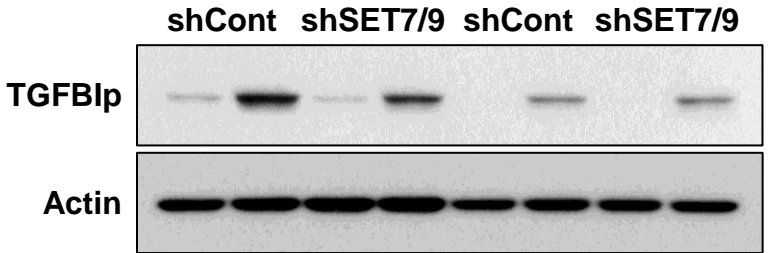

D

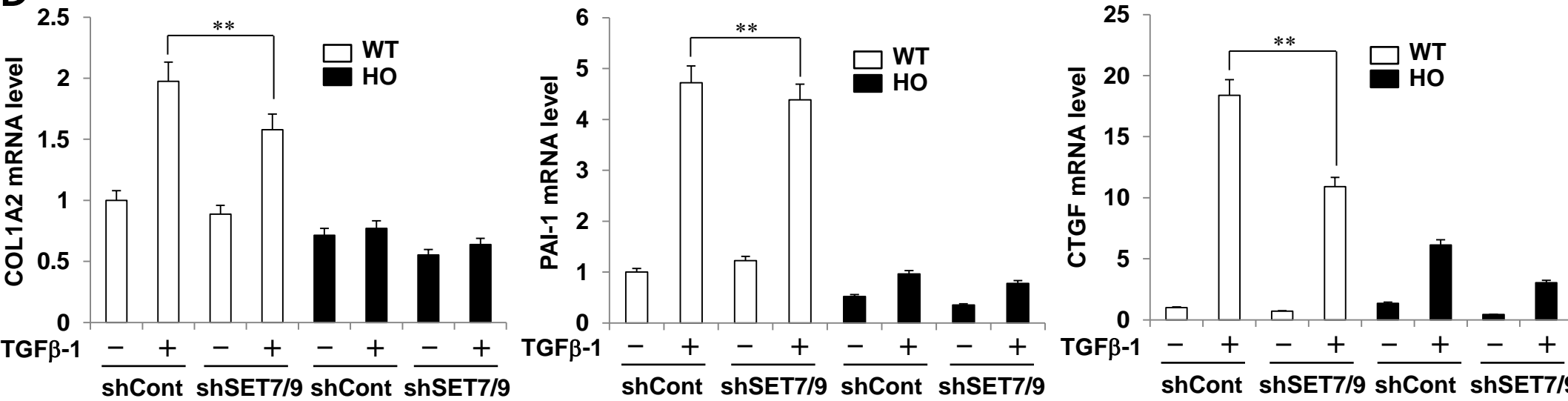

Supplementary Fig. 4

Chip:

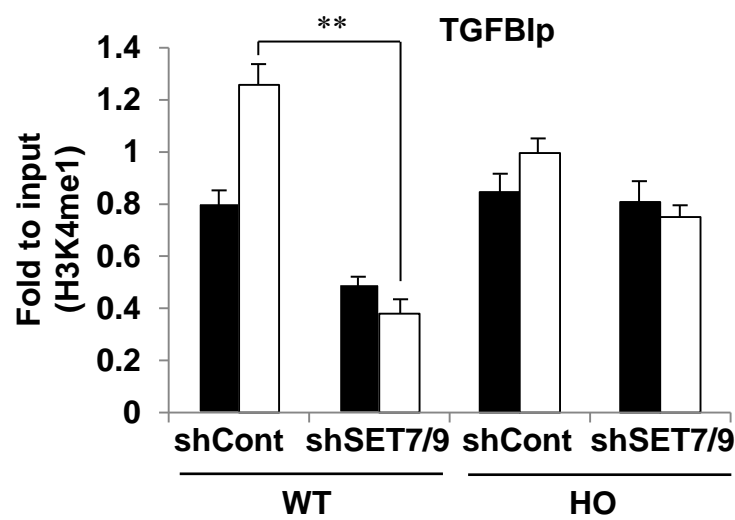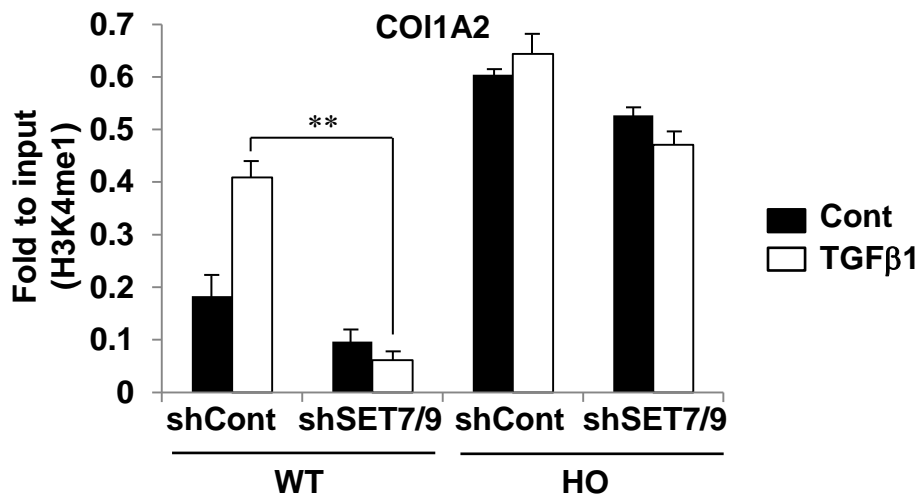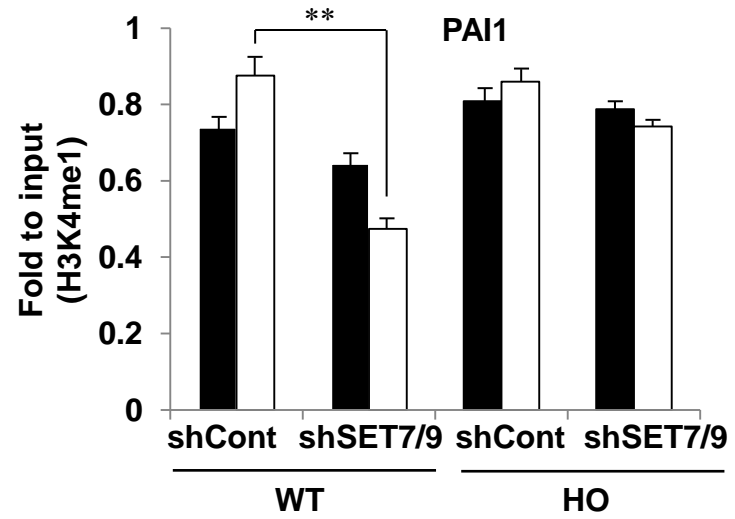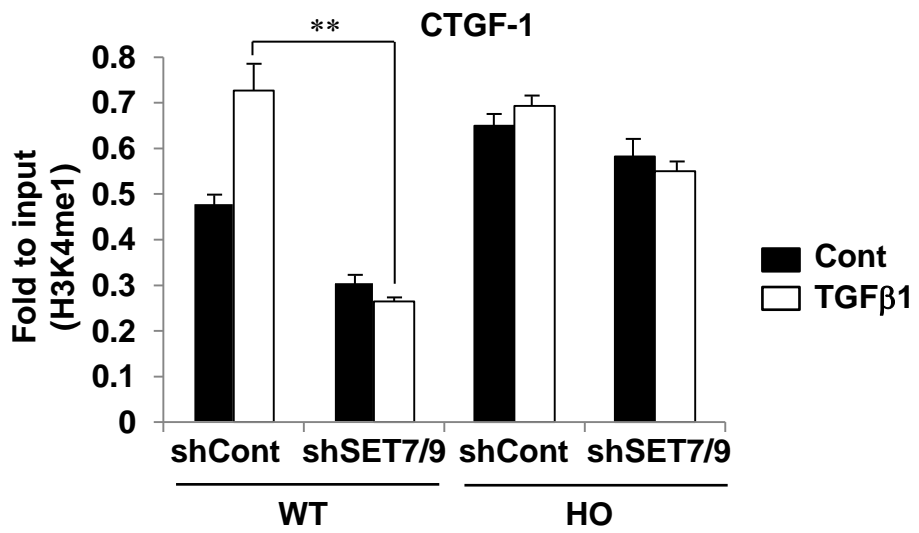

Supplement: Additional file 1: Figure S1. — TGFBIp transcription was high in wild-type cells compared with that in GCD2 corneal fibroblasts and was related to H3K4me3 levels. (A) H3K4me3 levels at the Smad binding elements of TGFBIp gene promoters in wild-type, GCD2-heterozygous, and GCD2-homozygous corneal fibroblasts. ChIP assays were performed with H3K4me3 antibody. (mean ±SE). (B) mRNA levels of TGFBIp in corneal fibroblasts were determined by RT-qPCR. (C, D) Protein levels of TGFBIp in cell lysates or culture supernatants were determined by western blot. Figure S2. TGFb1 had a minor effect on H3K27me3 and H3K9me3 modification on the TGFBIp and ECM-associated gene promoters. Bar graphs showing H3K27me3 (A) and H3K9me3 (B) levels on the indicated gene promoters in control and TGFb1 (5 ng/ml)-stimulated corneal fibroblasts. ChIP assays were performed with H3K27me3 and H3K9me3 antibodies. (mean ±SE; **P < 0.01 vs. control, n = 3). Figure S3. SET7/9 knockdown attenuated the TGFb1-induced expression of TGFBIp and ECM-associated genes. Wild-type and GCD2-homozygous corneal fibroblasts were infected with SET7/9 or control shRNA lentivirus. After puromycin selection, SET7/9, H3K4me1, TGFBIp, and actin protein levels were analyzed by western blot (A) and infected cells were stimulated with TGFb1 (5 ng/ml) for 8 h, and mRNA and protein levels of TGFBIp were analyzed by RT-qPCR (B) and western blot (C). mRNA levels of ECM associated genes were analyzed by RT-qPCR (D). (mean ±standard error (SE); **P < 0.01 vs. control, n = 3). Figure S4. SET7/9 knockdown attenuated TGFb1-induced increases in H3K4me1 levels at the promoters of TGFBIp and ECM-associated genes. SET7/9 or control shRNA lentivirus infected cells were stimulated with TGFb1 (5 ng/ml) for 8 h, and H3K4me1 levels at the indicated gene promoters were analyzed. ChIP assays were performed with H3K4me1 antibody. (mean ± SE); **P < 0.01 vs. control, n = 3). [file 12920_2015_151_MOESM1_ESM.pdf]
